# Product Control and Insight into Conversion of C6 Aldose Toward C2, C4 and C6 Alditols in One‐Pot Retro‐Aldol Condensation and Hydrogenation Processes

**DOI:** 10.1002/open.202100023

**Published:** 2021-05-04

**Authors:** Yingshuang Hui, Yulu Zhan, Wenrong Hou, Lou Gao, Yahong Zhang, Yi Tang

**Affiliations:** ^1^ Department of Chemistry Shanghai Key Laboratory of Molecular Catalysis and Innovative Materials Laboratory of Advanced Materials, Collaborative Innovation Centre of Chemistry for Energy Materials Fudan University 200433 postcode is missing Shanghai city is missing P. R. China

**Keywords:** Biomass, aldose, C4 alditol, ethylene glycol, one pot

## Abstract

Alcohols have a wide range of applicability, and their functions vary with the carbon numbers. C6 and C4 alditols are alternative of sweetener, as well as significant pharmaceutical and chemical intermediates, which are mainly obtained through the fermentation of microorganism currently. Similarly, as a bulk chemical, C2 alditol plays a decisive role in chemical synthesis. However, among them, few works have been focused on the chemical production of C4 alditol yet due to its difficult accumulation. In this paper, under a static and semi‐flowing procedure, we have achieved the product control during the conversion of C6 aldose toward C6 alditol, C4 alditol and C2 alditol, respectively. About C4 alditol yield of 20 % and C4 plus C6 alditols yield of 60 % are acquired in the one‐pot conversion via a cascade retro‐aldol condensation and hydrogenation process. Furthermore, in the semi‐flowing condition, the yield of ethylene glycol is up to 73 % thanks to its low instantaneous concentration.

## Graphical abstract

**The product control** of C6 aldose toward C6, C4 and C2 alditols have been achieved, which have depended on the well‐matched between retro‐aldol condensation and hydrogenation process driving by temperature, H_2_ pressure and injection modes.

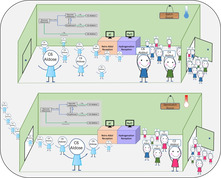

## 1 Introduction

Biomass‐derived alditols, including sorbitol, mannitol, xylitol, erythritol and others, could be acquired when aldehyde in sugars is reduced to hydroxy group.^[1]^ Similar to most of sugars, they could offer a wide range of sweetness. However, excessive intake of sugar, as all know, will cause human pancreatic islet dysfunction, diabetes, obesity and other diseases.^[2]^ Fortunately, not only can sugar alcohols be substitutes for traditional sugar to satisfy people‘s desire for sweet without causing obvious changes in blood sugar and insulin, but also they have less calorigenic properties.^[3]^ In addition, alditols with low carbon numbers, e. g. 1,2‐propylene glycol (1,2‐PG) and ethylene glycol (EG), as essential platform molecules, are widely used in cosmetic, food and pharmaceutical industry to produce various value‐added derivatives.^[4]^ Among them, C4 alditol is not only considered as a zero‐calorie sweetener but also as a potential chemical for production of C4 chemicals, such as butadiene, 1,4‐butanediol, tetrahydrofuran and other butanediols which are consumed in large scale and used in many fields.^[5]^ Dean et al. reported the successful deoxy dehydration to highly stereospecific olefin from C4‐C6 sugar alcohols catalyzed by methyltrioxorhenium using another alcohol as solvent.^[6]^

Currently, sugar alcohols mostly come from the fermentation broth of microorganisms and the hydrogenation of aldose.^[7]^ For example, sorbitol and mannitol can be obtained by hydrogenating glucose and mannose.^[8]^ The hydrogenation of sugar or biomass are usually catalyzed by some support catalyst based on noble metal such as Ru, Pd and Pt.^[9]^ Perrard et al. have achieved complete conversion of glucose hydrogenation over a Ru catalyst loaded on activated carbon with a sorbitol selectivity of 99.2 %.^[10]^ And xylitol can be hydrogenated by its corresponding sugar xylose.^[11]^ 1,2‐PG and EG can be prepared from the hydration of ethylene oxide and propylene oxide derived from petroleum cracking.^[4]^ Erythritol, the most marketable sweet substitute, almost comes from the fermentation of glucose.^[12]^ At present, there are two chemical processes to synthesize it, the one is first mixing acetylene and formaldehyde to obtain 2‐butene‐1,4‐diol, and then oxygenating it into erythritol.^[13]^ The other leverages an industrial process of starch or cellulose that contains acid and alkali treating, then oxidization by periodate and hydrogenation by nickel catalyst under high temperature and high pressure.^[14]^

In the last decades, hydrolysis of cellulose has attracted much attention. Scientists have been able to obtain glucose from cellulose at a high yield. Fukuoka's group reported for the first time the ability of using heterogenous catalysts to depolymerize cellulose into sugar alcohols most of which was sorbitol.^[15]^ They supplied a promising method producing polyols and essential value‐added chemicals from cellulose in the presence of a large amount of hydroxy groups. Zhang et al. developed a route to acquire 61 % yield of EG from cellulose over Ni−W/C.^[16]^ Palkovits et al. found that cellulose could be converted into C4−C6 sugar alcohols with a total yield of 81 %, catalyzed by Ru/C combined with heteropoly acid H_4_SiW_12_O_40_ under the conditions of 433 K and 5 MPa of H_2_.^[17]^ Though the sugar alcohol yield of 81 % is of high level, the yield of erythritol is less than 6 %, and the rest of high yield is mostly contributed by sorbitol and mannitol.

Herein, we studied one‐pot retro‐aldol and hydrogenation process of C6 aldose toward C2−C6 alditols under the static and semi‐flowing conditions, and explored the control factor influencing the product distribution of C2, C4 and C6 alditols. Under optimum conditions, the total yield of C4 and C6 sugar alcohols can exceed 60 % with the premise of C4 alditol yield of 20 % in the one‐pot system, whereas the yield of EG can be even up to 73 % in the semi‐flowing condition. These results provide the possibility of dynamical regulation of product distribution during the production of alditols from biomass derived sugars.

## 2 Results and Discussion

### 2.1 Product Control of C6, C4 and C2 Polyols in One‐Pot Reaction System

**Scheme 1:**
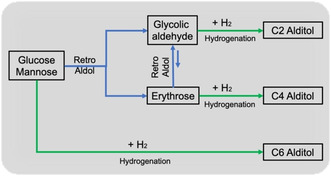
Conversion routes of C6 aldose toward various alditols.

As Scheme 1 shows, C6 aldoses can be directly converted into C6 alditol by the hydrogenation process. They can also produce C4 alditol and EG via a cascade retro‐aldol and hydrogenation process. C4 alditol is the hydrogenation product of the intermediate tetrose during retro‐aldol process of C6 aldose. However, accompanied with its hydrogenation, the further retro‐aldol process of tetrose toward glycolic aldehyde (GA) competitively occurs. This results in the difficulty in getting C4 alditol with high yield from C6 aldose. Obviously, for accumulating C4 alditol, the well matching between the production of tetrose by retro‐aldol process of C6 aldose and its hydrogenation is required. Hence, we screened the catalyst types and their ratios as well as the reaction temperature and pressure to explore the key factors in product control of C6 aldose conversion.

Table 1 shows the ability on hydrogenation of different aldoses in the presence of different noble metal catalysts. It can be found that, among three catalysts, Ru/C exhibits the highest hydrogenation efficiency, which is in line with the previous reports.^[18]^ Besides, it is clear that the hydrogenation of GA and C6 aldose can well perform with high conversion and high selectivity. However, the selectivity of C4 alditol is just lower than 50 % during the hydrogenation of erythrose (ERO), even though in the presence of Ru/C. Obviously, the high instability of ERO during the hydrogenation process further aggravates the difficulty in gathering of C4 sugar alcohol.

**Table 1:** Hydrogenation of C2−C6 aldose over catalysts in aqueous phase.

Sub.	Cat.	Conv. [C mol %]	Y. [C mol %]			Detect. carbon [C mol %]
			C6 alditol	C4 alditol	C2 alditol	
GLU	Ru/C	99.5^[a]^	98.5	0.6	0.2	99.8
	Pd/C	37.5^[a]^	3.9	4.6	0.9	71.9
	Pt/C	33.9^[a]^	4.8	3.4	0.8	75.1
ERO	Ru/C	>99.9^[a]^	–	32.9	8.3	41.2
		>99.9^[b]^	–	48.8	1.2	50.0
	Pd/C	60.0^[b]^	–	2.8	1.6	44.4
	Pt/C	64.5^[b]^	–	4.3	1.7	41.5
MAN	Ru/C	>99.9^[a]^	94.3	0.5	0.1	94.9
GA	Ru/C	>99.9^[a]^	–	6.5	90.4	96.9

Reaction condition: [a]. Equimolar substrate (2.22 mmol) and 50 mg of catalyst were added into 40 mL of water and the reaction was performed at 160 °C and 2 MPa of H_2_ for 1 h. [b] The reaction was performed at 170 °C and 3 MPa of H_2_ for 1 h.

According to our previous reports,^[19]^ tetrose could be attained with the yield of 30 % in retro‐aldol condensation process of C6 aldoses firstly. Taking the above selectivity of C4 alditol in the hydrogenation of ERO into account, theoretically, only less than C4 alditol yield of 15 % can be obtained. However, our experimental results demonstrate that the actual yield of C4 alditol is less than 5 % by two‐step method due to the high instability of tetrose. This means that a one‐pot process of retro‐aldol and hydrogenation process rather than a two‐step process have to be adopted to obtain C4 alditol with high yield. If tetrose in situ produced by retro‐aldol process of C6 aldose can be converted in time by hydrogenation, it is expected to improve the yield of C4 alditol. In view of this, we studied the role of product distribution of C6, C4 and C2 polyols in the one‐pot retro‐aldol and hydrogenation process of C6 aldose and the analyses and identifications of the various products are showed in Figure S1 and Figure S2.

It was reported that many Mo‐ and W‐series oxides or carbides and their salts could catalyze the splitting of C−C bond, which was mainly due to their Lewis acidities.^[20]^ Therefore, several kinds of W‐based and Mo‐based catalysts, i. e. ammonium tungstate (AT), ammonium metatungstate (AMT), ammonium paratungstate (APT) and amine phosphomolybdate (APM), were used as retro‐aldol catalysts to perform the tandem conversion of glucose (GLU) toward C2−C6 alditols with Ru/C. As shown in Table 2, W‐based catalysts display better catalytic performance than Mo‐based catalyst in retro‐aldol process. For C4 sugar alcohol accumulation, the reaction system catalyzed by AT and Ru/C gets the highest carbon yield, i. e. 17.5 %. These results also indicate, as expected, that C4 alditol can be better accumulated by one‐pot process. The consuming of the intermediate product tetrose and the generating of C4 alditol consecutively pull the equilibration of two tandem reactions so that one‐pot synthesis is more appropriate to produce C4 sugar alcohol.

**Table 2:** The product distribution and detectable carbon during the conversion of GLU in the presence of different retro‐aldol catalysts and Ru/C.

Retro‐aldol Cat.	Conv. [C mol %]	Y. [C mol %]			Detect. Carbon [C mol %]
		C6 alditol	C4 alditol	C2 alditol	
PM	55.1	7.7	1.8	7.1	61.4
APT	96.6	61.7	14.1	20.8	99.9
AMT	92.1	37.5	12.9	36.8	95.1
AT	96.6	51.0	17.5	26.5	98.4

Reaction condition: 400 mg of GLU, 50 mg of Ru/C and equimolar W/Mo‐based catalysts were added into 40 mL of water and the reaction was performed at 160 °C and 2 MPa of H_2_ for 2 h.

The ratio of the two used catalysts in this tandem reaction was studied to explore its influence on the product distribution. It shows that the retro‐aldol process of C6 aldoses is accelerated with the increasing amount of AT, which leads to a decreasing yield of C6 sugar alcohol and an increasing C2 alditol (Table 3).

**Table 3:** Conversion of C6 aldoses with different ratios of catalysts in aqueous phase.

Sub.	Ratio of AT:Ru/C [mg:mg]	Conv. [C mol %]	Y. [C mol %]			Detect. Carbon [C mol %]
			C6 alditol	C4 alditol	C2 alditol	
GLU	40 : 50	99.9	78.6	8.6	10.3	98.1
	60 : 50	96.9	51.9	14.7	25.3	96.7
	80 : 50	97.1	32.7	13.1	39.5	90.5
	100 : 50	97.5	44.4	15.2	31.1	95.6
MAN	40 : 50	96.3	64.5	13.3	17.3	99.7
	60 : 50	98.3	30.7	15.6	38.8	91.0
	80 : 50	97.9	37.8	19.0	34.1	96.5
	100 : 50	99.3	21.4	14.7	41.8	84.0

Reaction condition: 400 mg of GLU or MAN, 50 mg of Ru/C and different dosages of AT were added into 40 mL of water and the reaction was performed at 170 °C and 3 MPa of H_2_ for 2 h.

However, the yield of C4 alditol reaches the highest one and then decreases with the increasing ratio of AT:Ru/C. For example, the highest yield of C4 alditol could be obtained when the ratio of AT:Ru/C is 80 : 50 (mg:mg) (Table 3). On the one hand, the accumulation of C4 alditol requires the rapid retro‐aldol process to avoid excessive direct hydrogenation of hexose. On the other hand, the rapid retro‐aldol rate will lead to the formation of EG from ERO. Moreover, the low hydrogenation selectivity of ERO further limits the accumulation of C4 sugar alcohol. That's why few works were published discussing the acquisition of C4 sugar alcohol from hexose conversion. It is worth noting that, in the fermentation industry, a number of studies have focused on the deliberate co‐production of C4 alditol with another compound of interest such as C6 alditol.^[21]^ Therefore, taking the high yields of C4 and C4+C6 sugar alcohols into consideration, the 80 : 50 (mg:mg) ratio of AT:Ru/C is adopted.

**Figure 1:**
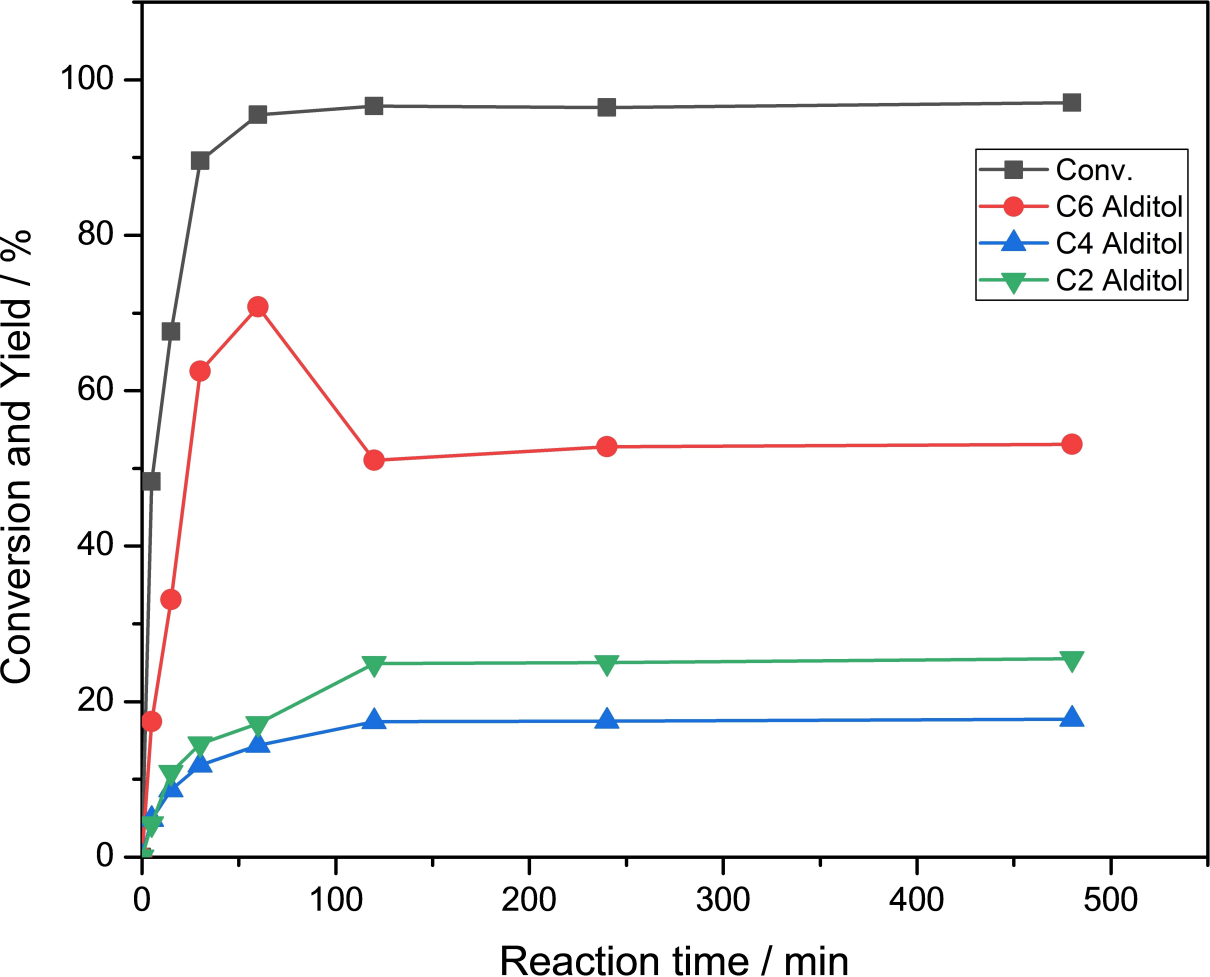
Conversion of GLU and its product distribution with the increasing reaction time under 2 MPa of H_2_ and 160 °C.

Besides, when the ratio of AT:Ru/C is fixed at 80 : 50 (mg:mg), the conversion of GLU and the corresponding product distribution had been investigated with the change of reaction time. GLU has been consumed mostly after 1 h and the yields of the various products reach their ceiling after the reaction proceeded for 2 h (Figure 1). More specifically, the yield of sorbitol increases rapidly in the first hour, then decreases slowly owing to its hydrogenolysis to glycols in the second hour, and finally keeps constant with the extending time.^[22]^ At the same time, C4 alditol and other sugar alcohols increase steadily and then settle after 2 h. Hence, the reaction time of 2 h is the best time point to obtain the target product. Additionally, C4 alditol and other alditols are steady and no hydrogenolysis or reduction reactions occur, implying the high stability of these alditols.

**Figure 2:**
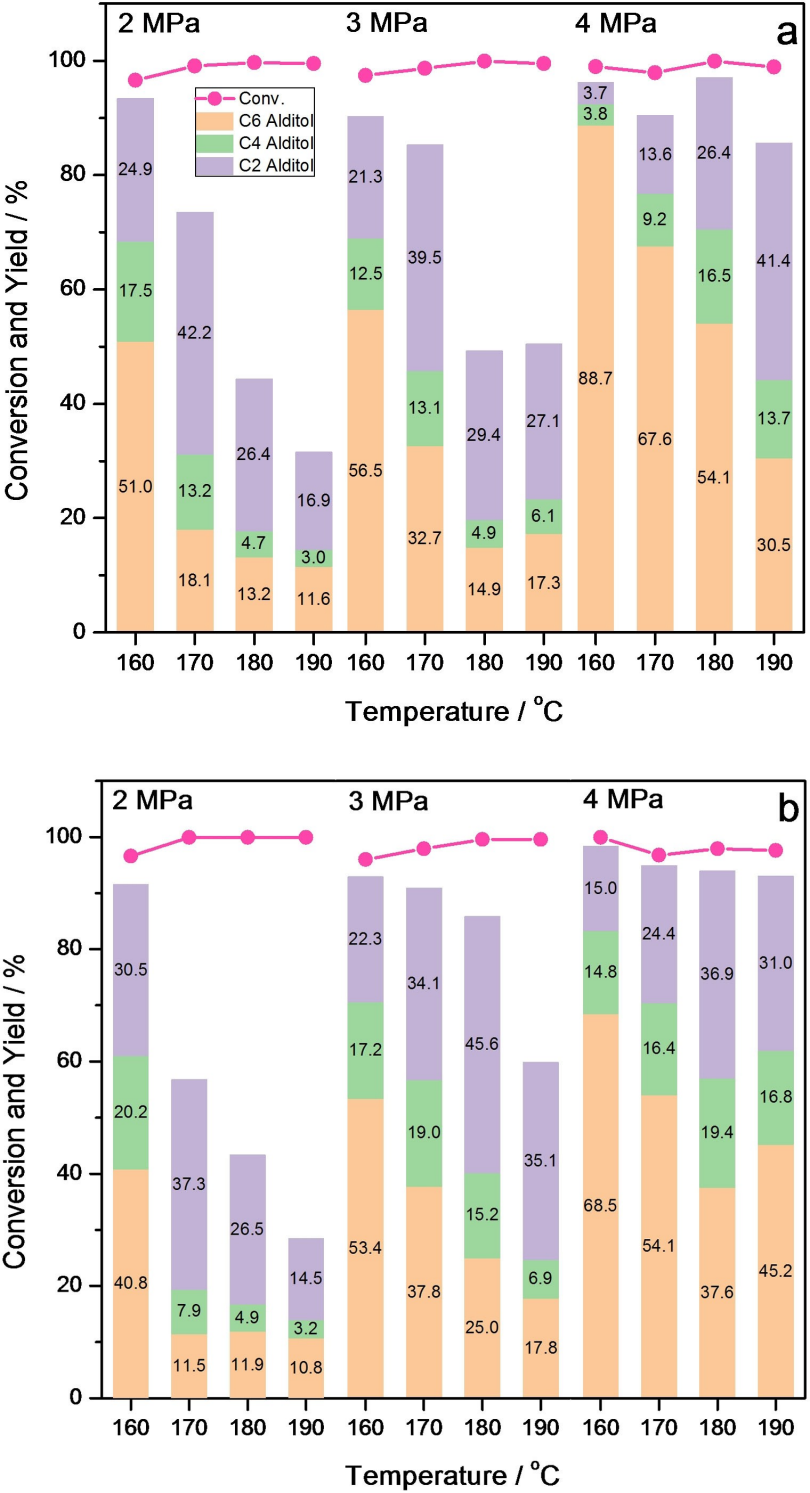
The distribution of products in the conversion of (a) GLU and (b) MAN under different H_2_ pressures at 160–190 °C.

Furthermore, the reaction temperature and H_2_ pressure have been tuned to maximize the yields of both C4 and C4+C6 sugar alcohols and the results were listed in Figure 2. The yield of C6 sugar alcohols dramatically decreases with the increasing reaction temperature, while the low H_2_ pressure results in a low detectable carbon at high temperature. This means that high temperature contributes to the retro‐aldol process of C6 aldoses rather than hydrogenation. The fast retro‐aldol process and slow hydrogenation process bring about low detectable carbon owing to the instability of the retro‐aldol products. However, a high H_2_ pressure can accelerate the hydrogenation process of all the substrates including hexoses (GLU or MAN) and their retro‐aldol products (ERO and GA). The fast hydrogenation can convert timely instable ERO and GA into stable C4 and C2 polyols and assure a high detectable carbon. The different influences of reaction temperature in the retro‐aldol and hydrogenation process can be explained by their thermodynamics analyses. According to Scheme 1, the retro‐aldol processes of C6 aldoses toward ERO and GA as well as ERO toward GA are process of the particle increase, which means that their entropy changes (ΔS) are positive. Thus, the increasing reaction temperature will lead to a more negative free energy change according to ΔG=ΔH−T ⋅ ΔS, which is responsible for the right shift of retro‐aldol process with the increase of reaction temperature. However, contrary to the retro‐aldol process, the hydrogenation processes of C2−C6 aldoses definitely are the process of negative entropy change (ΔS<0). The free energy change will become more positive when the reaction temperature rises, which implies that high temperature shows an adverse effect on the hydrogenation process. For the H_2_ pressure, the high H_2_ pressure can promote the shift of hydrogenation reaction from left to right according to Le Châtelier's Principle. For example, the yield of sorbitol increases from 55.0 % to 88.7 % when the pressure of H_2_ raises from 2 MPa to 4 MPa at 160 °C. Therefore, for the purpose of the accumulation of C4+C6 sugar alcohols with high yield and the high detectable carbon, a low reaction temperature and H_2_ pressure or a high reaction temperature and H_2_ pressure are required. The total yield of C4+C6 sugar alcohols can finally reach 60 %–70 % and the yield of C4 alditol can be higher than its theoretic one (15 %) under the above two conditions.

### 2.2 Product Control of C6, C4 and C2 alditols in semi‐continuous reaction system

In one‐pot reaction system, by carefully controlling reaction temperature and H_2_ pressure, we can either obtain the highest yield for C4 alditol (20.2 %) and C6 alditol (88.7 %) or achieve over 60 % of the C4 and C6 sugar alcohols co‐production. However, as the final product of retro‐aldol and hydrogenation of C6 aldoses, whatever the reaction conditions are, the carbon yield of EG is lower than 50 %. Taking competition between retro‐aldol condensation and hydrogenation of aldose, it is expected that reducing the instant concentration of substrate could prompt the shift of retro‐aldol process towards final product. Hereinafter, we tried to use the semi‐continuous method to decrease the instantaneous concentration of substrate in the system, i. e. the substrate with a certain concentration was injected into solution containing catalysts with a certain feeding rate.

**Figure 3:**
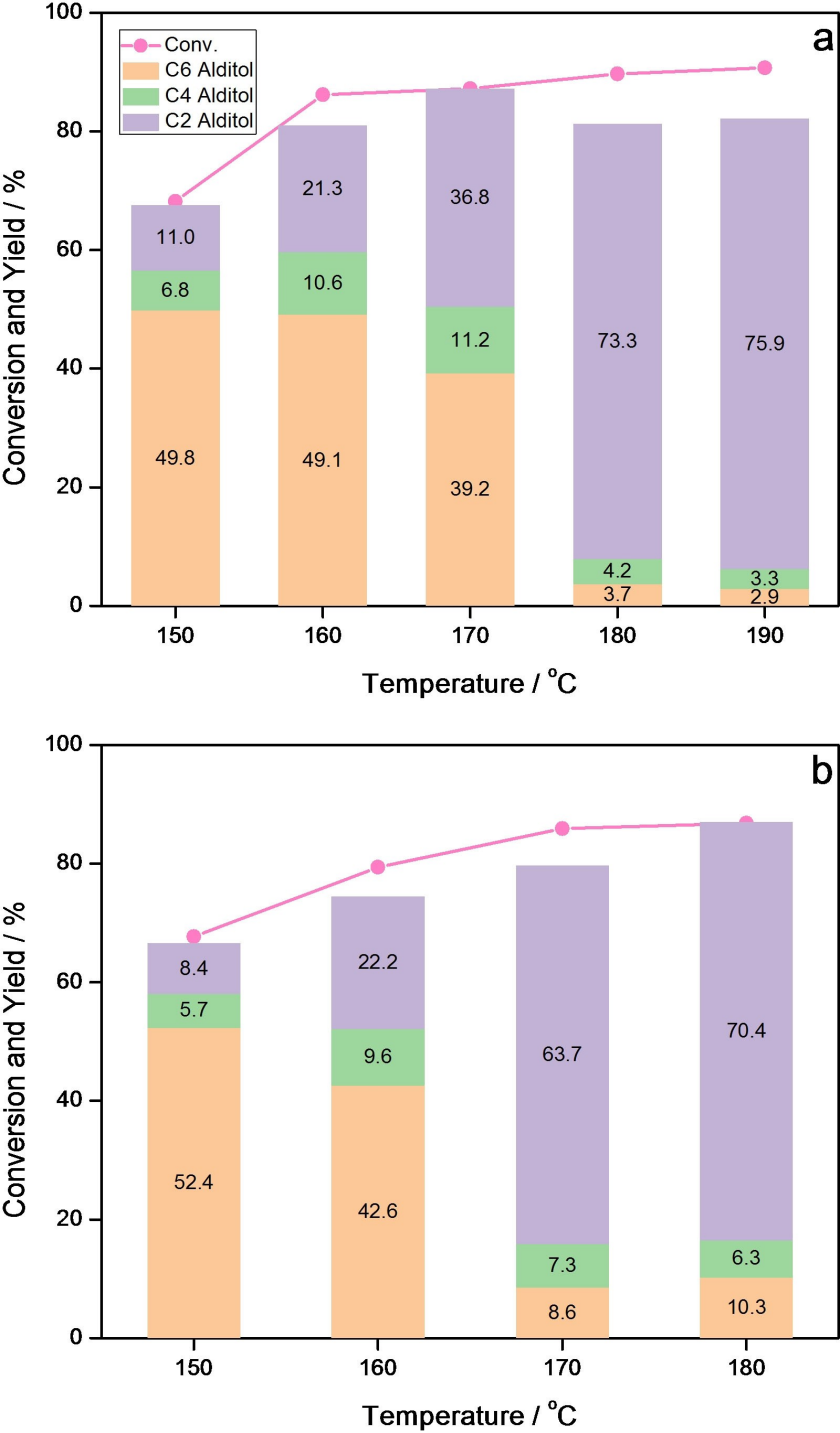
Conversion of GLU at different temperatures under (a) 2 MPa and (b) 4 MPa of H_2_ in semi‐continuous reaction system, keeping injecting with 15 g/L glucose solution.

As shown in Figure 3, the yield of C6 alditol is still high under low reaction temperature in spite of low instantaneous concentration of hexose. However, with the increasing reaction temperature, the yield of C6 alditol dramatically decreases and that of EG greatly increases. For example, under 2 MPa of H_2_ pressure, the yield of C6 polyol decreases from 49.8 % to 2.9 % with the changing reaction temperature from 150 °C to 190 °C while EG rises from 10.9 % to 75.9 % (Figure 3a and Figure S3). This is much higher than the yield of EG reported by the present literatures on GLU conversion toward EG.^[23]^ Moreover, the yield of EG also increases rapidly with the increase of reaction temperature even though the pressure of H_2_ is increased to 4 MPa. These results further confirmed that higher temperature is conductive to the retro‐aldol process rather than hydrogenation. However, when the pressure of H_2_ is decreased to 1 MPa, all the detectable carbon retains low under all the temperature ranges (Figure S4). Obviously, the H_2_ pressure of 1 MPa is not enough to hydrogenate the hexose and the products of its retro‐aldol condensation in time under any reaction temperature. Interestingly, the yield of C4 alditol reaches a maximum value at a moderate reaction temperature (Figure 3). Here we can draw an analogy, i. e., taking the reaction system as a balance, C6 alditol and C2 alditol sit at the two ends of the balance, and the temperature is the weight mastering the balance. The higher the temperature is, the more thorough the reaction shifts to C2 alditol. Herein, C4 alditol is like the fulcrum of the balance in the continuous reaction, which reaches a maximum when the productions of C6 and C2 alditol are well‐matched in rate.

In our study, the differences in product distribution of semi‐continuous reaction root in the change of the instantaneous concentration of substrate in the process of feeding fluid. To obtain the optimal condition, the conversion of hexoses under feed solution with different concentrations were studied. As illustrated in Table 4, in a particular range of substrate concentration, the yield of EG does not change dramatically, which results from the steady instantaneous concentration. However, when the concentration of substrate rises to a certain amount, the detectable carbon drops sharply, and the yields of EG are also suppressed. Moreover, the effect of the flowing rate on the EG yield displays the similar trend to that of the substrate concentration (Figure S5), and the feeding rate of 0.2 mL/min is finally adopted.

**Table 4:** The conversion of feed solution with different concentrations and types of sugar.

Sub.	C_sub_. [g/L]	Conv. [C mol %]	Y. [C mol %]				Detect. carbon [C mol %]
			C6 alditol	C4 alditol	C2 alditol	C3 alditol [1,2‐PG]	
GLU	10	89.9	8.2	4.9	70.8	– (3.7)	97.7
	15	96.7	3.7	4.2	73.3	– (5.3)	89.7
	20	91.1	3.5	4.7	65.4	– (5.5)	87.9
	25	90.5	2.2	4.8	58.3	– (6.1)	81.0
MAN	10	90.4	9.9	6.7	71.5	– (1.8)	99.5
	15	90.4	6.7	5.9	73.3	– (2.8)	96.8
	20	91.8	3.2	5.9	70.6	– (1.1)	90.0
	25	93.1	2.3	4.7	55.5	– (4.3)	73.7
SUC	15	88.7	3.9	2.3	44.8	9.6 (21.4)	94.4
MAL	15	84.2	15.1	5.6	34.4	– (2.7)	80.6

Reaction condition: 80 mg of AT and 50 mg of Ru/C were put into the reactor in advance, and performed under 180 °C and 2 MPa of H_2_, keeping injecting 15 g/L different sugar solution with 0.2 mL/min for 100 min.

Similarly, this semi‐continuous process can also be applied to some disaccharides (Table 4). For the conversion of disaccharide toward EG, there are three tandem steps, i. e. hydrolysis, retro‐aldol condensation and hydrogenation. AT with Lewis acidity is expected to break β‐glycosidic bond. Obviously, fast hydrolysis and retro‐aldol process are achieved for sucrose, and so a low C6 alditol yield (3.9 %) and a high EG yield (44.8 %) can be observed. Moreover, a considerable amount of C3 products (∼30.0 %) containing alditol and 1,2‐propylene glycol (1,2‐PG) is detected since sucrose (SUC) is composed of glucose and fructose. Different from GLU, fructose tends to produce two molecules of C3 compounds via a retro‐aldol condensation. In the same way, maltose (MAL) can also be hydrolyzed into two molecules of glucose and undergo a series of reactions to acquire products. However, probably because of its slow hydrolysis process, only 34.4 % yield of EG can be obtained while the yield of C6 alditol rises to 15.1 %.

## 3 Conclusion

This work has achieved the product control of C6 aldose toward C6, C4 and C2 alditols under the static and semi‐flowing conditions, respectively. It is found that a high temperature is in favour of the retro‐aldol process of C6 aldoses whereas a high H_2_ pressure can promote the hydrogenation process. By the well‐matched between retro‐aldol condensation and hydrogenation process, we can obtain 20 % of C4 alditol and more than 60 % of C4 and C6 sugar alcohols in the one‐pot system. In the semi‐flowing condition, the yield of EG can be up to 73 % thanks to its low instantaneous concentration of substrate. Such flexibility on the production of products can achieve a fast anticipation on varying market demands and prices of the produced polyols such as ethylene glycol, 1,2‐propanediol, glycerol and erythritol.

## Experimental Section

### Materials

Sucrose (SUC, 97 %), Maltose (MAL, 97 %), Mannose (MAN, 99 %), D‐Threitol (97 %) and Ammonium Metatungstate (AMT, 99.5 %) were purchased from Shanghai Aladdin Biochemical Technology Co., Ltd. Erythrose (ERO, 75 %), meso‐Erythtirol (99 %), Erythrulose (ERU, 85 %), 1,2‐propylene glycol (1,2‐PG, 98 %), Ammonium Phosphomolybdic (APM, AR) and Ammonium Paratungstate (APT, 99.5 %) were purchased from Shanghai Macklin Biochemical Technology Co., Ltd. Glycolaldehyde dimer (GA, 98 %), Glucose (GLU, 99.5 %), Fructose (99 %) and Ethylene glycol (EG, 99.8 %) were purchased from Sigma‐Aldrich. Mannitol (98 %) and Sorbitol (98 %) were purchased from Sinopharm Chemical Reagent Company. Ammonium tungsten oxide (AT, 99.9 %) was purchased from Alfa Aesar. Deionized water was produced by a laboratory water purification system. All other reagents were commercially available and were used as received.

The hydrogenation catalysts used in the investigation were Ru/C (5 % Ruthenium on activated carbon, Maclin), Pd/C (5 % Palladium on activated carbon, Aladdin) and Pt/C (5 % Platinum on activated carbon, Aladdin). XRD patterns of these catalysts indicated their small metal sizes (Figure S6).

### Catalytic Experiments

The following procedure was used for the experiments shown above. In a typical one‐pot experiment, substrate, catalyst and deionized water (40 mL) were added in a stainless‐steel autoclave (Parr Instrument Company, 100 mL). Then, the reactor was closed and flushed 5 times with H_2_. After applying the desired H_2_ pressure, stirring was started (400 rpm) and the reactor was heated to the desired temperature. The starting time of the reaction was determined as the point when the reactor reached the desired temperature (approx. 30 min). To stop the reactions, the reactors were allowed to cool to room temperature with cooling water (approx. 10 min).

In a typical semi‐continuous experiment, a certain concentration of C6 aldose solutions was prepared. After reaching the desired temperature and pressure, the substrate solution was injected using pump with the feeding rate of 0.2 mL/min for 100 min into the reactor containing Ru/C (50 mg), AT (80 mg) and 20 mL deionized water which were put into batch in advance, and finally keep reacted for another 20 min.

### Product Analysis

The reaction solution of 100 μL was taken out and diluted to 1000 μL with deionized water. The sample were analyzed on high performance liquid chromatography (HPLC, Shimadzu Corporation) equipped with refractive index detector (RID). The reaction products were separated using Bio‐Rad Aminex HPX‐87H column at 35 °C with 8.0 mM H_2_SO_4_ aqueous solution as the mobile phase at the flow of 0.6 mL/min. Meanwhile, the reaction products were separated using COSMOSIL sugar‐D column (4.6 mm lD×300 mm) at 40 °C with an aqueous solution containing 90 % acetonitrile as the mobile phase at the flow of 0.8 mL/min. Before being injected into HPLC the samples needed to be filtered through a micro syringe filter. The retention time of detectable sugars and sugar alcohols by Bio‐Rad Aminex HPX‐87H column were as follow: GLU (8.7 min), MAN (9.4 min), FRU (9.6 min), ERO (11.4 min), GA (11.7 min), ERU (11.8 min), and C4 alditol can be detected by COSMOSIL sugar‐D column at 11.9 min. Each product, as well as reactant, were calibrated by using its standard at different concentrations at their specific retention times. High‐resolution mass spectra of reaction products were recorded in Bruker McriOTOF II mass spectrometer.

For the preparation of alditol from aldoses, conversions of substrates and carbon yields of products were calculated as follows:

$Conv.\;\left(C\;mol\%\right)=\left[1-\frac{Mole\;of\;substrate\;in\;the\;product}{Initial\;mole\;of\;substrate}\right]\times 100\%$

${Y.\;}_{C6\;Alditol}\;\left(C\;mol\%\right)=\frac{Mole\;of\;sorbitol\;or\;mannitol\;in\;the\;product}{Initial\;mole\;of\;hexose}\times 100\%$

${Y.}_{C4\;Alditol}\;\left(C\;mol\%\right)=\frac{2}{3}\times \frac{Mole\;of\;C4\;alditols\;in\;the\;product}{Initial\;mole\;of\;hexose}\times 100\%$

${Y.}_{C2\;Alditol}\;\left(C\;mol\%\right)=\frac{1}{3}\times \frac{Mole\;of\;ethyene\;glycol\;in\;the\;product}{Initial\;mole\;of\;hexose}\times 100\%$

$Detect.\;carbon\;\left(C\;mol\%\right)=$ $\left[1-Conv.\;+{Y.}_{C6\;Alditol}+{Y.}_{C4\;Alditol}+{Y.}_{C2\;Alditol}+{Y.}_{others}\right]\times 100\%$

## Conflict of interest

The authors declare no conflict of interest.

## Supporting information

As a service to our authors and readers, this journal provides supporting information supplied by the authors. Such materials are peer reviewed and may be re‐organized for online delivery, but are not copy‐edited or typeset. Technical support issues arising from supporting information (other than missing files) should be addressed to the authors.

Supplementary
