# Supplementary material for: Product Control and Insight into Conversion of C6 Aldose Toward C2, C4 and C6 Alditols in One‐Pot Retro‐Aldol Condensation and Hydrogenation Processes
Source: ChemistryOpen. 2021 May 4;10(5):560–6. doi: 10.1002/open.202100023 (PMC8095293; doi:10.1002/open.202100023)

ChemistryOpen

Supporting Information

Product Control and Insight into Conversion of C6 Aldose Toward C2, C4 and C6 Alditols in One-Pot Retro-Aldol Condensation and Hydrogenation Processes

Yingshuang Hui, Yulu Zhan, Wenrong Hou, Lou Gao, Yahong Zhang,* and Yi Tang

Supporting Information (SI)

Figure S1. MS analysis results of the reaction solution from the conversion of C6 aldose.

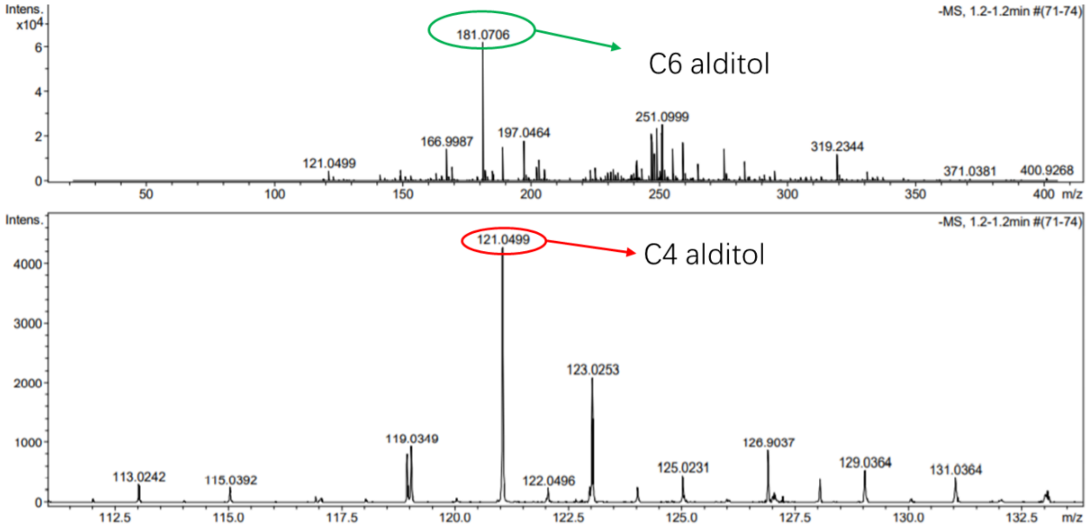

Reaction condition: 400 mg of MAN, 80 mg of AT and 50 mg of Ru/C were added into 40 mL of water and the reaction was proceeded under 170 °C and 3 MPa of H_2 for 2 h.

Figure S2. Representative HPLC-RID analysis results of reaction solution in one-pot process via using Bio-Rad Aminex HPX-87 column (a) and Sugar-D column (b). A = mannitol, B = sorbitol, C = ethylene glycol, D = erythritol.

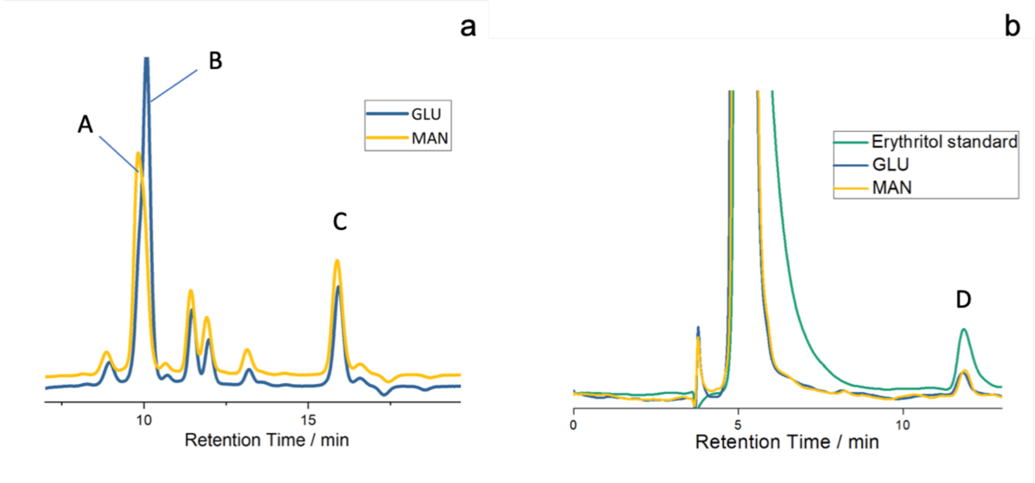

Reaction condition: 400 mg of GLU or MAN, 50 mg of Ru/C and 80 mg of AT were added into 40 mL of deionized water. The reaction was performed under 160 °C and 2 MPa of H_2 for 2 h.

Figure S3. Representative HPLC-RID analysis result of reaction solution in the semi-continuous process via using Bio-Rad Aminex HPX-87 column. A = glucose, B = mannose, C = mannitol, D = sorbitol, E = ethylene glycol.

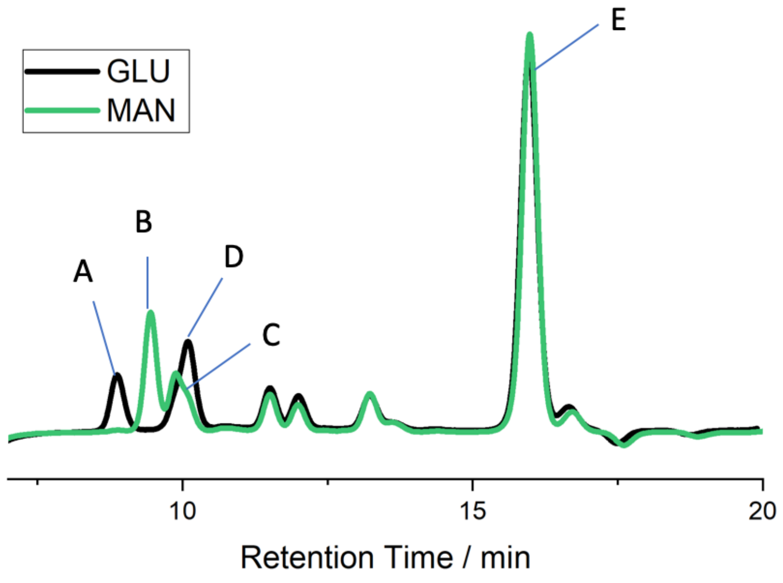

Reaction condition: 80 mg of AT and 50 mg of Ru/C were put into the reactor in advance, and performed under 180 °C and 2 MPa of H_2, keeping injecting 15 g/L GLU solution with 0.2 mL/min for 100 min and keep reacted for more 20 min.

Figure S4. Conversion of GLU at different temperatures under 1 MPa of H_2 in one-pot semi-continuous reactor.

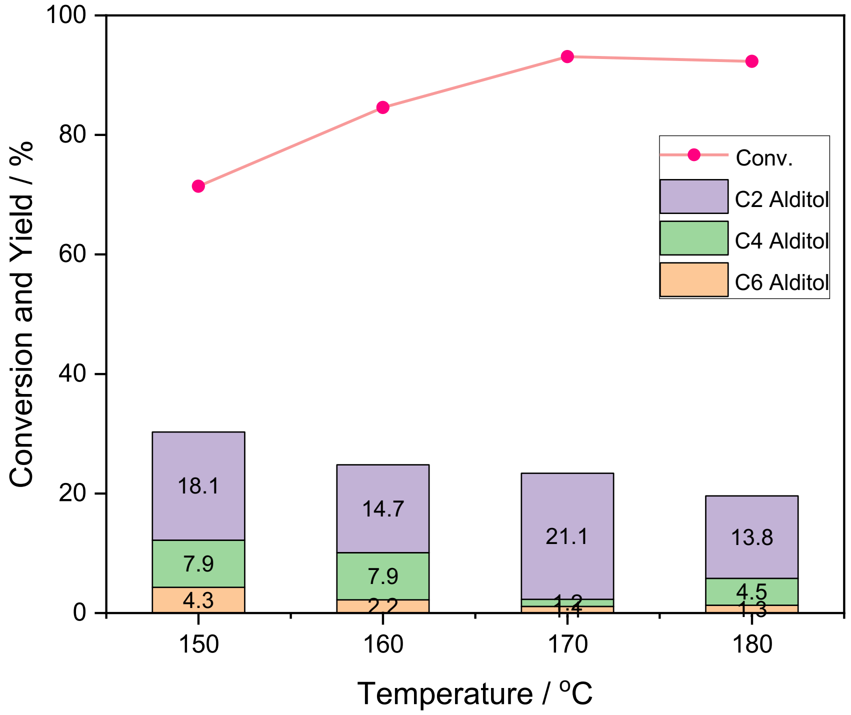

Reaction condition: 80 mg of AT and 50 mg of Ru/C were put into the reactor in advance, and performed at different temperatures and 1 MPa of H_2, keeping injecting 15 g/L glucose solution with 0.2 mL/min for 100 min and keep reacted for more 20 min.

Figure S5. Conversion of GLU with different feeding rates under 2 MPa of H_2 in one-pot semi-continuous reactor.

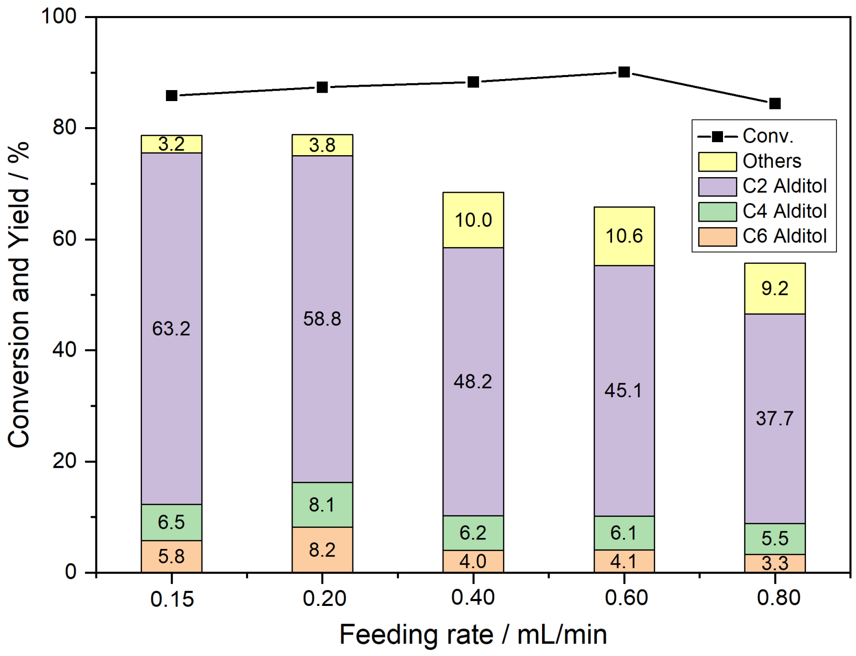

Reaction condition: 80 mg of AT and 50 mg of Ru/C were put into the reactor in advance, and performed at 170 °C and 2 MPa of H_2, keeping injecting 15 g/L glucose solution with different feeding rates.

Figure S6. Powder XRD patterns of Ru/C, Pd/C and Pt/C.

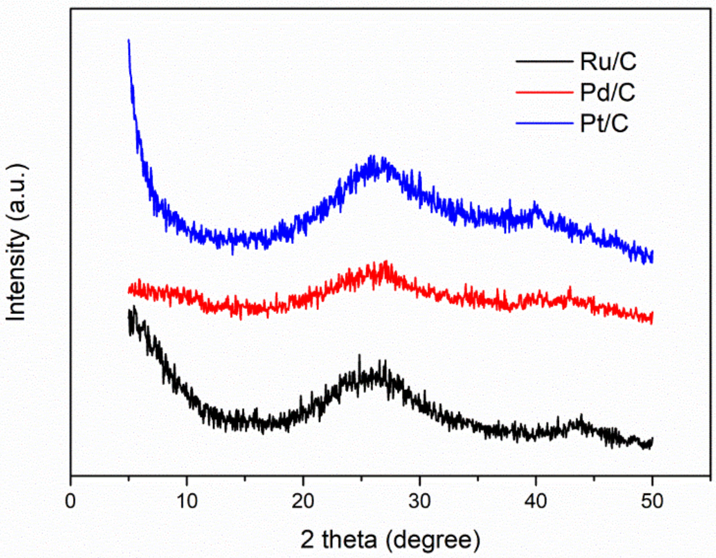

Supplement: Supplementary file 1 — Supplementary [file OPEN-10-560-s001.pdf]
